# Supplementary material for: SSR-Sequencing Reveals the Inter- and Intraspecific Genetic Variation and Phylogenetic Relationships among an Extensive Collection of Radish (Raphanus) Germplasm Resources
Source: Biology (Basel). 2021 Nov 30;10(12):1250. doi: 10.3390/biology10121250 (PMC8698774; doi:10.3390/biology10121250)
Supplement: Supplementary file 1 [file biology-10-01250-s001.zip › biology-1446288-supplementary Figures.pdf]

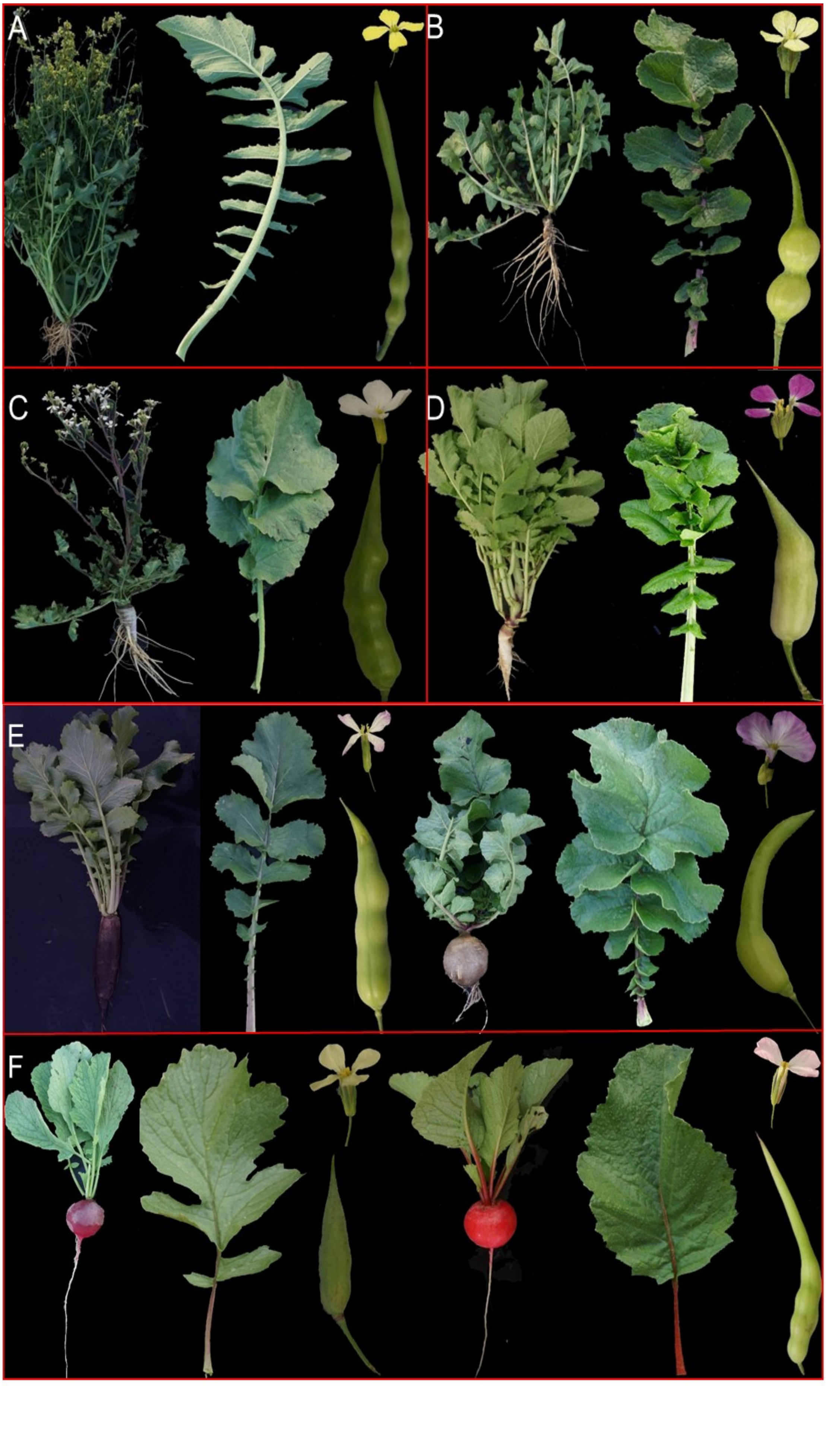
A
B
C
D
E
F

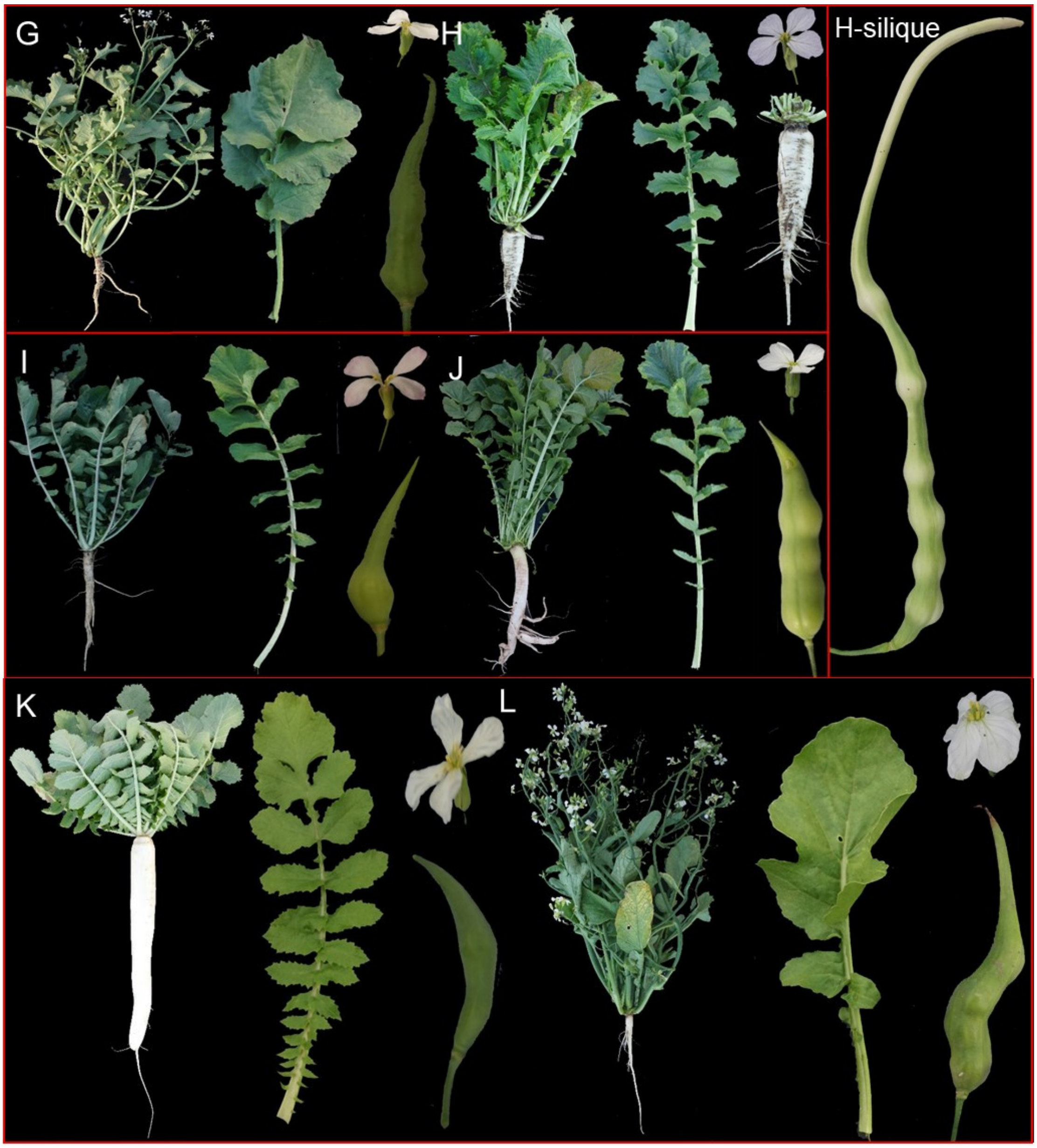
G
H
H-silique
I
J
K
L

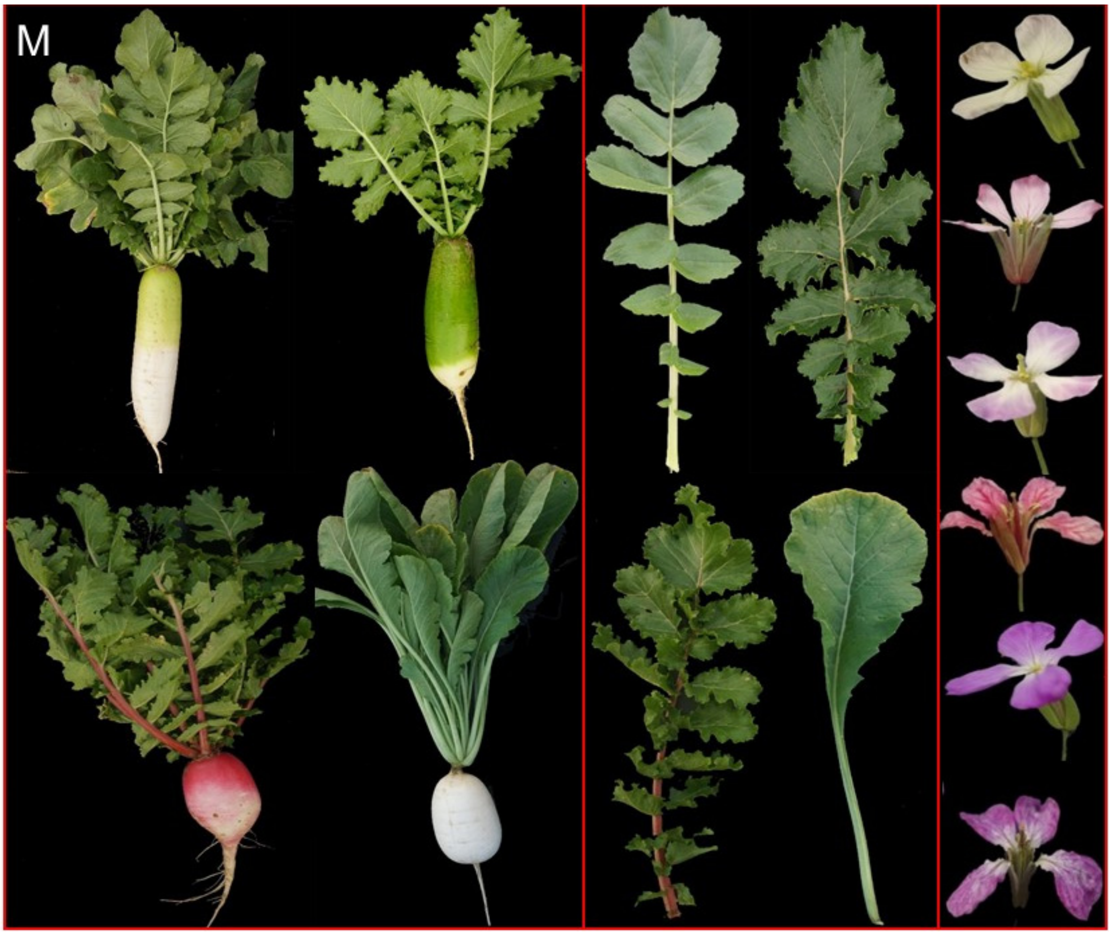

Figure S1: Characteristic images of ten radish categories. **European wild radish**: (A: *R. raphanistrum* subsp. *raphanistrum*. B: *R. raphanistrum* subsp. *landra*). C: **European primitive cultivated radish**. D: **European oil radish**. E: **black radish**. **F**: **European small radish**. G: **American wild radish**. H: **rat-tail radish**. I: **Japanese wild radish**. **Japanese and Korean big radish** (J: Japanese big radish; K: Korean big radish). L: **Chinese oil radish** and M: **Chinese big radish.**

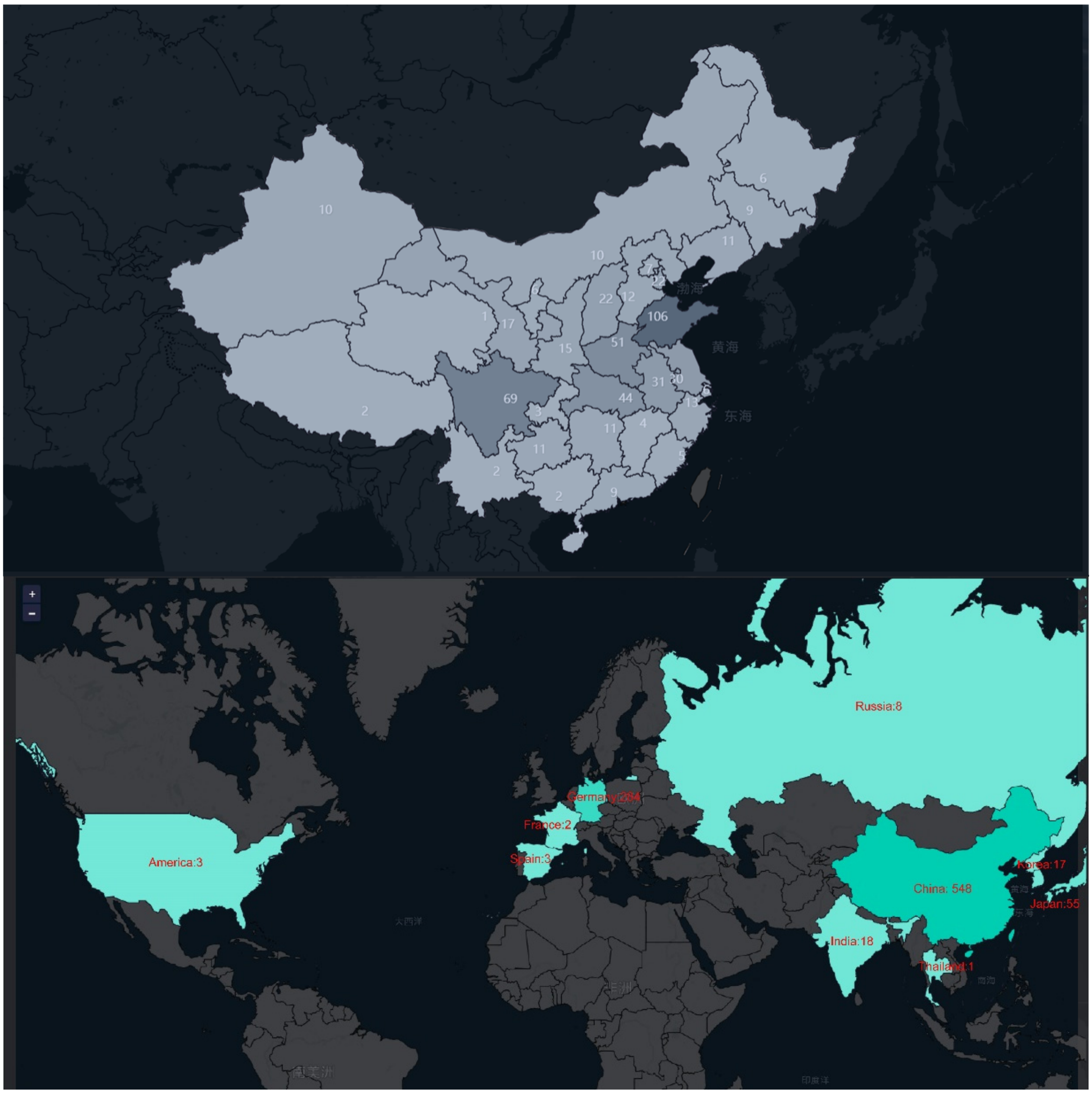

Figure S2 Distribution of 548 Chinese radish accessions (Chinese big radish and Chinese oil radish) in China and distribution of 939 radish accessions in world.

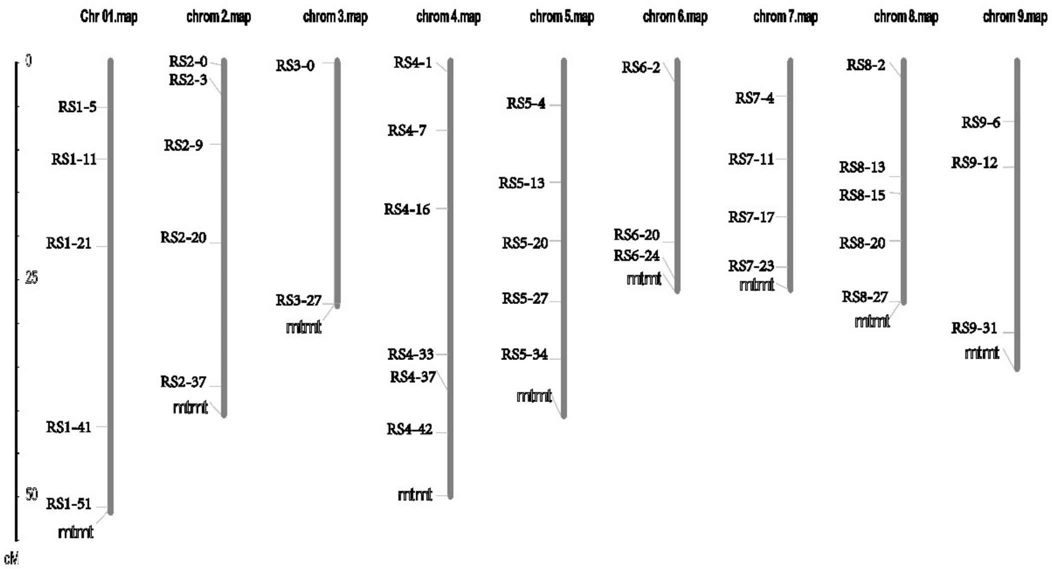

Figure S3 Distribution of 38 pairs of genomic SSRs across all 9 radish chromosomes.
