# Supplementary material for: SSR-Sequencing Reveals the Inter- and Intraspecific Genetic Variation and Phylogenetic Relationships among an Extensive Collection of Radish (Raphanus) Germplasm Resources
Source: Biology (Basel). 2021 Nov 30;10(12):1250. doi: 10.3390/biology10121250 (PMC8698774; doi:10.3390/biology10121250)
Supplement: Supplementary file 1 [file biology-10-01250-s001.zip › biology-1446288-supplementary Tables.pdf]

Table S1. Basic information of the 38 SSR primers used in this study.

Primer name	Forward. Primer	Reverse. Primer
RS1-5	CTGACCGTAATCCTTGAGCC	CCAACGACGGAGGAGATAGA
RS1-11	GGGAAGAAGAGGTTTGTGATT	AACGGAATGAAATAAAAGCG
RS1-21	TGACTGCAATTTGTTCTTCTCT	TCCAAAATGCTTCTTTTCAA
RS1-41	CAAGGAGGAAACCGTGAAAA	GGAAGTGATTTTTGCCTTGG
RS1-51	TTGTTGTTGCCTTGTAGGGA	CACACATGGAAGAACTCTCAGC
RS2-0	CTTCTACTTCTTTGTCGCCC	AGAAGAGGGAATCGGAAACT
RS2-3	AGGAATGGATTTTGGTGAAA	AGCAAGCAAGAAGAAGCAAT
RS2-9	CTTCTGTCCACGGATTACCT	TGAGGATGATGTTTCTGAATG
RS2-20	AGAGCTGGATTGAAAGATGG	CACTGCAGGAACTCCTTTCT
RS2-37	CGTCTTTTTGCAAATGCGT	CAAGATTTACTTACAACTTGTCATCG
RS3-0	AAAGGCCAAAGCTCCTCTTC	CGAGTTTCTTCTTGAAGGCG
RS3-27	GCAAATAATGAGATCGCCGT	GGCATCGTCAAGTCATTCCT
RS4-1	AGGATTGCCGTGATGAAGAC	TCCCTAGCAAAGTGACCAGC
RS4-7	CAAAAGCAGTGGAGTCAAGA	CTTGTTTTAGTTGGCTTTTGC
RS4-16	CAAACGATTGAATGCTGTTC	AGCTTTCTTGCTTTCGTCAT
RS4-33	TCCGACAAGAACATAAACCC	TTCCCTTTCACCACAGTTCT
RS4-37	CATTTTCTACGGCCAAGATT	TTTAAGGGTTCCTTTCTCCC
RS4-42	AGGCAAATAAAAGTCCCCAT	CCGATGATTCTTCTCCCTT
RS5-4	AGGCGTGAGTGGAGAAGAAA	AGCGCTTGACGATACCTCAT
RS5-13	AAGCGCTCGAAAGTTCTCT	GTTCCTAAAACCGCAGGTAA
RS5-20	AGAATCTCTCGTTCCAGACG	CAATAAACACGCCTCACAAA
RS5-27	TGTCATACCGTTCTGCATTT	GCTAAGCTACTCCTTGAGCG
RS5-34	GCTTCACCGGATAATTCAAC	GACTAAAAAGCAGAGCAGGG
RS6-2	TTTTCCAGAACTCCCAACAT	AGAAGAAGCTAAAAAGGGGC
RS6-20	GAAGGGATTAACGAGCTCAA	TCATAGCTTTCATGTGGCAT
RS6-24	CAGTGCGTGAGACTATTTGG	GAGAAGAAGACGGCAGAGAG
RS7-4	GAATTCTCACTCTGTTCGCC	AGAGCTTACGGATTTGGATG
RS7-11	GTGATTTAAAAATCCCGCAC	ACCTCGAGTGTCTTCACCAT
RS7-17	CATTCACCAATCTCTAGCCA	AATACGAAGGATGAGGTGGA
RS7-23	CCTTACGGAGAAGAAAACACA	CCTCGATGATTTCAAAATGG
RS8-2	AGAAGAAACAACCCATCACC	TCAAGTCTCGTATCCCAATG
RS8-13	CCTCGTCACTGTCGTAATCA	GGACGGTCGAAGAGAAATAA
RS8-15	GTCACATGACCCATCTTTGA	AATGACGGTGTCAGAGGTTT
RS8-20	GGTTCATGCATACAAGTGACA	AGCTTTTAAGCAAGACGCTG
RS8-27	CGGGGATAGAGGAGAGATTT	TCCACCCTTTCTCTTTCATC
RS9-6	GGCAAATCCGTCAAGTTCAT	CCGGGTTTTGATTTTGAAGA
RS9-12	AAGCACGAATACCCAAGTTC	CTCTTCTTTTCTCAGCTGGC
RS9-31	ATCTGGTCGTAACGGTGGAG	TTTAACAACCGGAGGTGGTC

Table S2. Genetic diversity of all radish accessions reflected by different indexes based on SSR-Seq with 38 pairs of genomic SSR primers.

Locus	Na	Ne	Ho	He	Nei	I	Locus	Na	Ne	Ho	He	Nei	I
RS1-5	5	2.93	0.27	0.66	0.66	1.18	RS5-27	7	3.16	0.26	0.68	0.68	1.42
RS1-11	13	5.23	0.37	0.81	0.81	1.80	RS5-34	12	4.63	0.44	0.78	0.78	1.74
RS1-21	11	3.95	0.74	0.75	0.75	1.57	RS6-2	9	3.60	0.30	0.72	0.72	1.43
RS1-41	8	2.60	0.20	0.62	0.61	1.28	RS6-20	11	2.84	0.40	0.65	0.65	1.31
RS1-51	6	2.40	0.18	0.58	0.58	0.97	RS6-24	16	2.36	0.26	0.58	0.58	1.45
RS2-0	9	3.02	0.27	0.67	0.67	1.42	RS7-4	10	2.55	0.22	0.61	0.61	1.16
RS2-3	14	4.77	0.39	0.79	0.79	1.93	RS7-11	14	4.48	0.48	0.78	0.78	1.93
RS2-9	10	4.09	0.29	0.76	0.76	1.62	RS7-17	14	4.09	0.68	0.76	0.76	1.61
RS2-20	10	3.54	0.28	0.72	0.72	1.55	RS7-23	15	2.78	0.26	0.64	0.64	1.49
RS2-37	3	2.05	0.10	0.51	0.51	0.86	RS8-2	18	2.19	0.26	0.54	0.54	1.48
RS3-0	6	3.00	0.22	0.67	0.67	1.26	RS8-13	17	4.26	0.47	0.77	0.77	1.99
RS3-27	11	2.02	0.15	0.50	0.50	1.20	RS8-15	22	4.14	0.46	0.76	0.76	2.04
RS4-1	7	2.09	0.22	0.52	0.52	0.82	RS8-20	17	3.87	0.19	0.74	0.74	1.72
RS4-7	8	2.56	0.22	0.61	0.61	1.13	RS8-27	17	4.63	0.54	0.78	0.78	2.07
RS4-16	17	3.96	0.24	0.75	0.75	1.72	RS9-6	7	3.17	0.23	0.68	0.68	1.28
RS4-33	8	1.90	0.20	0.47	0.47	1.04	RS9-12	17	5.97	0.34	0.83	0.83	2.11
RS4-37	9	4.41	0.27	0.77	0.77	1.61	RS9-31	9	2.32	0.14	0.57	0.57	1.20
RS4-42	9	4.02	0.16	0.75	0.75	1.65	Mean	11.16	3.33	0.30	0.67	0.67	1.45
RS5-4	6	2.39	0.18	0.58	0.58	0.99	SD	4.39	1.04	0.14	0.10	0.10	0.35
RS5-13	14	2.00	0.26	0.5	0.50	1.04	Min	3	1.90	0.10	0.47	0.47	0.82
RS5-20	8	2.60	0.17	0.62	0.62	1.13	Max	22	5.97	0.74	0.83	0.83	2.11
